# Association between ratio for diameters of pulmonary artery to ascending aorta bifurcation in chest CT scan and number of involved vessels in coronary angiography

**DOI:** 10.1186/s13104-021-05459-1

**Published:** 2021-02-05

**Authors:** Iman Mohseni, Afshin Shiri, Simindokht Mojahedin

**Affiliations:** 1 Radiology Department, Iran University of Medical Sciences, Tehran, Iran; 2 Cardiology Department, Shahid-Beheshti University of Medical Sciences, Tehran, Iran

**Keywords:** Coronary artery disease, Imaging, CT scan, Angiography

## Abstract

**Objective:**

Coronary artery disease (CAD) is an important cause of mortality and morbidity, therefore, recognizing its severity and related factors is important. This study was performed to evaluate the association between ratio for diameters of pulmonary artery to ascending aorta bifurcation in chest CT scan and number of involved vessels in coronary angiography. In this observational cross-sectional comparative study, 110 patients who were under coronary angiography in Firoozgar Hospital in 2017 were enrolled, and the association between ratio for diameters of pulmonary artery to ascending aorta bifurcation in their chest CT scan and number of involved vessels in angiography were assessed.

**Results:**

In this study, number of involved vessels in angiography was related to PA/Ao ratio (*P* = 0.001) and further vessels were accompanied with higher ratio. It may be concluded that, a higher ratio for diameters of pulmonary artery to ascending aorta bifurcation in chest CT scan is related to higher number of involved vessels in coronary angiography, and it may have a predictive role.

## Introduction

Coronary Artery Disease (CAD) is recognized as the first cause of the mortality worldwide and also as the leading cause of morbidity and high economic costs [1–3]. It has shown an increasing trend in many countries including Iran with a raise percentage between 25 and 40 percent [4–7]. Also, there are multiple risk factors for coronary artery disease [8–14], which can even increase the severity of coronary artery disease [15–20]. Prompt diagnosis and treatment of disease can lead to decrease in mortality and mobidity [21]. Sudden cardiac death is considered as the main cause of the cardiac deaths presenting as early symptom [20]. The prevalence rate of coronary artery disease and angina pectoris among Iranian population is 21.8 and 10.7 percent, respectively [19]. Conventional diagnostic methods, especially in the patients with chest pain include the folowings: angiography (gold standard), echocardiography, stress echocardiography, scintigraphy, computed tomography angiography (CT-angiography), cardiac MRI, nuclear scan, and exercise test [22]. There are few non-invasive diagnostic methods applicable in asymptomatic patients. Pulmonary artery diameter in an imaging index and the association with aortic diameter in imaging are markers of pulmonary hypertension [4, 5]. Pulmonary hypertension is related to heart failure with ejection fraction preservation, morbidity, and mortality [6, 7], especially in chronic obstructive pulmonary disease (COPD) patients [8, 9]. An association has been oberved between pulmonary hypertension and pulmonary artery to ascending aorta diameter in these patients in previously performed studies [10, 11], as a prognostic factor in cardiovascular diseases [12, 16]. However, the diagnostic role is not considered yet. Since majority of diagnostic methods for CAD are invasive; in this study, the association between ratio for diameters of pulmonary artery to ascending aorta bifurcation in chest CT scan and number of involved vessels in angiography was determined.

## Main text

### Materials and methods

In this observational cross-sectional descriptive-comparative study, 110 consecutive patients who were under coronary angiography (with maximal time interval of three-month with chest CT scan in the same center) were enrolled for cardiovascular symptoms in Firoozgar Hospital, Tehran, Iran, in 2017. The study was approved by local ethical committee. Also, the collected data were recorded in checklists including demographic, clinical, and imaging variables.

All CT scans were performed without contrast and with Siemens SOMATOM Scope 16-Slice CT Scanner device at Firoozgar Hospital, Tehran, Iran.almost all ct scans use a range of 110–130 kVp depending on patient body habitus and mAs/ref adjusted proportionally between 70–80. Total dose length product (DLP) of procedure measured 180 mGycm on average.

**Fig. 1:**
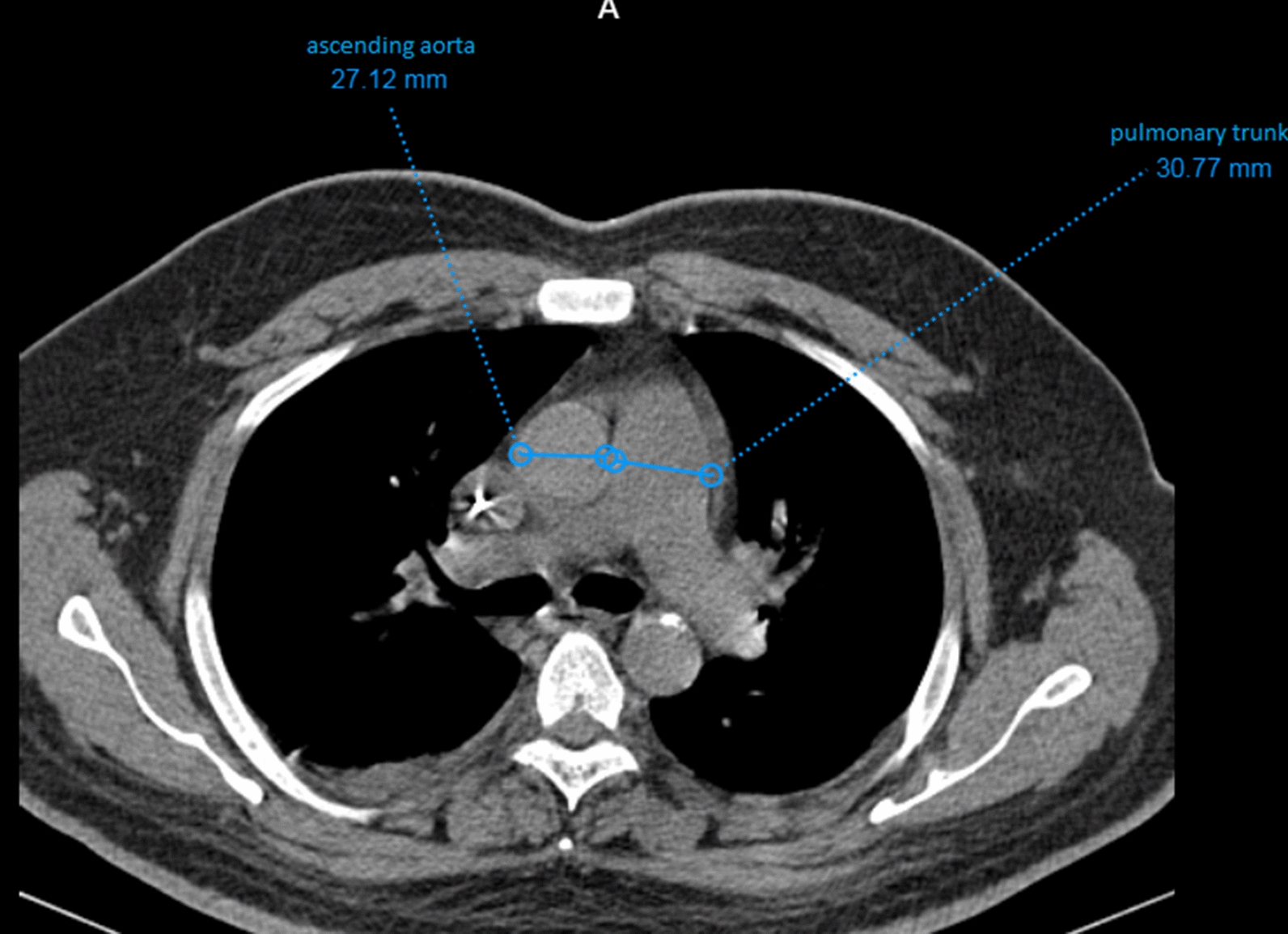
measurement is performed at the level of pulmonary artery bifurcation

The results of chest CT scan were reported by two expert radiologists, and the mean measurements (as shown in Fig. 1) were reported. The association between the ratio for diameters of pulmonary artery to ascending aorta bifurcation in their chest CT scan and number of involved vessels in angiography were assessed.

Data analysis among 110 subjects was done using SPSS version 19.0 software. The utilized tests for performing comparisons in this study were Chi-Square and Analysis of variance (ANOVA), and the P values less than 0.05 were considered as statistically significant.

### Results

In this study, the mean (standard deviation) age was 65.4 (12.8) years old. Also, there were 63 male subjects (57.3%). Majority of the subjects had 2-vessel and 3-vessel involvements (Additional file 1).

Mean (standard deviation) pulmonary artery diameter, ascending aorta, and the related ratio was 30 (7.0), 32 (4.7), and 0.94 (0.16), respectively. Older age was related (*P* = 0.004) to further involved vessels in coronary angiography (Table 1). There was no significant association between the number of involved vessels in coronary angiography and gender (*P* = 0.161) (Additional file 2).

**Table 1:** Comparative number of involved coronary vessels by age

Age (year)		
Number of vessels	Mean	Std. deviation
NVD	52.50	16.662
SVD	63.20	9.317
2VD	67.82	12.825
3VD	67.31	11.528

*SVD* single vessel disease, *2VD* 2 vesel disease, *3VD* three vessel disease, *NVD* no vessel disease; *Std* standard

Data are presented as mean standard deviation.

As shown in Table 2, aorta diameter was not related to the number of involved vessels in coronary angiography (*P* = 0.514). However, higher pulmonary artery diameter (*P* = 0.026) and the calculated ratio (*P* = 0.001) were related to higher number of involved vessels in coronary angiography in the patients.

**Table 2:** Association between involved vessels in coronary angiography and chest CT scan measurements

Number of vessels		PA diameter (mm)	Aorta diameter (mm)	Ratio
NVD	Mean Std. deviation	24.90 2.378	31.30 4.373	0.8071 0.11687
SVD	Mean Std. deviation	28.25 7.525	31.75 5.369	0.8619 0.12387
2VD	Mean Std. deviation	30.53 5.311	32.92 5.359	0.9487 0.14577
3VD	Mean Std. deviation	31.64 8.192	31.45 3.704	0.9990 0.17785

*PA* pulmonary artery, *SVD* single vessel disease, *2VD* 2 vesel disease, *3VD* three vessel disease, *NVD* no vessel disease; *Std* standard

Data are presented as mean standard deviation

### Discussion

In this study, the alternative diagnostic role of ratio for diameters of pulmonary artery to ascending aorta bifurcation in chest CT scan was assessed as a non-invasive method, which showed a good diagnostic value beside a previously approved prognostic role in this era. This matter can also help in early diagnosis of CAD in the patients undergone chest CT scan for any other non-cardiac reason, and subsequently the mortality and morbidity in the communities by CAD can be decreased. In this study, it was found that, a higher ratio is related to further involved vessels in coronary angiography.

We introduce the PA/Ao ratio as a non-invasive alternative diagnostic and screening method in patients who undergone CT scan for other reasons, nevertheless it is not applicable as an independent diagnostic tool such as an ECG or exercise test yet and more studies should be designed to evaluate the diagnostic power, sensitivity and specificity of this test compared to the other non-invasive methods mentioned.

A cohort study by Well et al. [11] demonstrated that, a higher PA/Ao ratio is related to the increased mortality in COPD patients, as an independent factor, especially in severe cases. However, our cross-sectional design in the current study could not assess the outcomes in the patients. Kavakus et al. [13] reported that, PA/Ao ratio is a marker of pulmonary hypertension in the patients with Heart failure with preserved ejection fraction (HFPEF), and is also related to disease's outcome. In our study, same association was indirectly seen with the severity of involvement in coronary angiography.

Campton et al. [15] reported that, PA/Ao ratio is normally higher than 1 during childhood period. However, as shown in our study, higher measurements in adults were abnormal and considered as an index for severity of disease.

Antlanger et al. [16] reported PA/Ao ratio as a prognostic marker that is related to the severity of disease, and is also associated with some risk factors such as diabetes, atrial fibrillation, right-sided heart failure, Brain natriuretic peptide (BNP), and low Glomerular filtration rate (GFR), especially in women. Nevertheless, in our study, gender was not related to the severity of involvement in the subjects. Also, it has never been studied as an independent alternative diagnostic method for coronary heart disease screening, which this study proves.

According to a mentioned study by Well et al. The PA/Ao ratio is an independent mortality risk factor in COPD patients. This ratio has also been correlated with coronary heart disease risk factors according to antlanger et al. study. One can hypothesis increased mortality rate of COPD patients which was attributed to PA/Ao ratio may also be due to coronary artery involvement, in order to find a more certain result more study focusing on COPD patients is required.

Nasrullah et al. [17] reported that, ascending aorta diameter in children is routinely higher than adults, and it should be remembered in the studies with wide ranges. Also, Truong et al. [18] reported that, pulmonary artery diameter is more in male subjects compared to female cases. In our study, only age was related to a higher severity of involvement; however, there was observed no effect for gender.

It may be concluded that, a higher ratio for diameters of pulmonary artery to ascending aorta bifurcation in chest CT scan is related to higher number of involved vessels in coronary angiography, which may have a predictive role even in the subjects who have incidental findings in chest CT scan due to other causes.

## Limitation

Further studies with larger sample sizes and multi-center sampling, and also by considering possible confounding role for cardiovascular risk factors are required to attain more definite results in this era.

## Supplementary Information

**Additional file 1.** Number of involved vessels in angiography.

**Additional file 2.** Comparative number of involved coronary vessels by gender

## Data Availability

The datasets used and/or analysed during the current study available from the corresponding author on reasonable request.

## Abbreviations

CAD: Coronary artery disease
COPD: Chronic obstructive pulmonary disease
CT-angiography: Computed tomography angiography
DLP: Dose length product
ANOVA: Analysis of variance
HFPEF: Heart failure with preserved ejection fraction
BNP: Brain natriuretic peptide
GFR: Glomerular filtration rate
PA: Pulmonary artery
SVD: Single vessel disease
2VD: 2 Vesel disease
3VD: Three vessel disease
Std: Standard
NVD: No vessel disease
