# Supplementary material for: Association between ratio for diameters of pulmonary artery to ascending aorta bifurcation in chest CT scan and number of involved vessels in coronary angiography
Source: BMC Res Notes. 2021 Feb 5;14:49. doi: 10.1186/s13104-021-05459-1 (PMC7866663; doi:10.1186/s13104-021-05459-1)
Supplement: Supplementary file 2 — Additional file 2. Comparative number of involved coronary vessels by gender [file 13104_2021_5459_MOESM2_ESM.docx]

Number of involved vessels in angiography
